# Low phonon energies and wideband optical windows of La_2_O_3_-Ga_2_O_3_ glasses prepared using an aerodynamic levitation technique

**DOI:** 10.1038/srep45600

**Published:** 2017-03-30

**Authors:** Kohei Yoshimoto, Atsunobu Masuno, Motoi Ueda, Hiroyuki Inoue, Hiroshi Yamamoto, Tastunori Kawashima

**Affiliations:** 1 Materials & Advanced Research Laboratory, Nikon Corporation, 2-15-3 Konan, Minato-ku, Tokyo 108-6290, Japan; 2 Graduate School of Science and Technology, Hirosaki University, 3 Bunkyo-cho, Hirosaki, Aomori 036-8561, Japan; 3 Institute of Industrial Science, The University of Tokyo, 4-6-1 Komaba, Meguro-ku, Tokyo 153-8505, Japan

## Abstract

*x*La_2_O_3_-(100 − *x*)Ga_2_O_3_ binary glasses were synthesized by an aerodynamic levitation technique. The glass-forming region was found to be 20 ≤ *x* ≤ 57. The refractive indices were greater than 1.92 and increased linearly with increasing *x*. The polarizabilities of oxide ions were estimated to be 2.16–2.41 Å^3^, indicating that the glasses were highly ionic. The glasses were transparent over a very wide range from the ultraviolet to the mid-infrared region. The widest transparent window among the oxide glasses was from 270 nm to 10 μm at *x* = 55. From the Raman scattering spectra, a decrease in bridging oxide ions and an increase in non-bridging oxide ions were confirmed to occur with increasing La_2_O_3_ content. The maximum phonon energy was found to be approximately 650 cm^−1^, being one of the lowest among oxide glasses. These results show that La_2_O_3_-Ga_2_O_3_ binary glasses should be promising host materials for optical applications such as lenses, windows, and filters over a very wide wavelength range.

Optical materials that are widely transparent from the ultraviolet (UV) to the infrared (IR) range are in great demand for many optical designs. In particular, UV–IR and visible–IR coaxial optical systems have been developed rapidly for multi-imaging cameras, vehicle video systems, endosomes, and biological microscopes in recent years. However, optical materials that are useful over wide wavelength ranges from the UV to the IR include fluoride crystals and glasses^1^. The critical problems of such fluorides are difficulties in their mass production and their poor chemical durability; thus, their practical applications are limited. Although oxide glasses are more practical and commonly used as optical components because of their high productivities and chemical durabilities, their optical windows are usually much narrower. For instance, conventional oxide glasses such as silicate and borate glasses generally have good UV–visible transparency, but their IR cut-off wavelength reaches at most 2–3 μm due to the large phonon energies of the network formers^2^.

Ga_2_O_3_-based glasses, on the other hand, possess good IR transparency in oxide glasses. Heavy metal gallate glasses, proposed by Dumbaugh in 1986, have attracted attention because of their longer IR cut-off edges of up to 8 μm^3^. In these glasses, however, the transparency in the UV to visible range is degraded due to strong s → p transitions in heavy metal cations such as Pb^2+^ and Bi^3+^. As for the alkali or alkali earth gallate glasses, Fukumi and Sakka investigated the structures of the most basic *R*_2_O- or *R*’O-Ga_2_O_3_ binary glasses (*R* and *R*’ denote alkali and alkali earth ions, respectively) using Raman^4^ and XRD^5^. Additionally, Kokubo *et al*. revealed the glass-forming regions, the optical transmittance, and the structures of various ternary gallate glasses such as (*R*_2_O or *R*’O)-Ta_2_O_5_-Ga_2_O^6^, (*R*_2_O or *R*’O)-Nb_2_O_5_-Ga_2_O_3_^7^, and (*R*_2_O or *R*’O)-TiO_2_-Ga_2_O_3_^8^. As for the rare-earth gallate system, however, there has only been report by Yajima *et al*., which described the glass formation and crystallization behaviors of 3*Ln*_2_O_3_-5Ga_2_O_3_ (*Ln* represents a rare-earth ion) compositions using an impact-quenching technique^9^. Very recently, Kidkhunthod *et al*. reported the glass formation of Pr_3_Ga_5_O_12_ glass by a levitation technique and investigated local structure around Pr and Ga atoms^10^.

Levitation techniques are useful to obtain bulk glasses by vitrification of materials with high melting temperatures and low glass-forming ability, such as rare-earth gallate binary gallate systems. Levitation is a containerless melting process in which the sample is levitated and melted by non-contact heating such as laser irradiation. This enables the suppression of heterogeneous nucleation from the container wall and promotes deep undercooling and vitrification of the melt. In recent years, Al_2_O_3_-11,12,13,14,15, TiO_2_-16,17,18,19,20, Nb_2_O_5_-21,22,23, and WO_3_-based^24^ glasses without any network formers have been discovered using this method. These “unconventional” glasses showed outstanding dielectric, optical, and mechanical properties as a result of the specific glass compositions and structures. In this study, we aimed to obtain glasses without devitrification in the simple La_2_O_3_-Ga_2_O_3_ binary system using a levitation technique and to clarify the fundamental physical properties. In addition, Raman scattering spectra were taken for structural analysis and evaluation of phonon energies of the glasses.

## Results and Discussion

**Figure 1:**
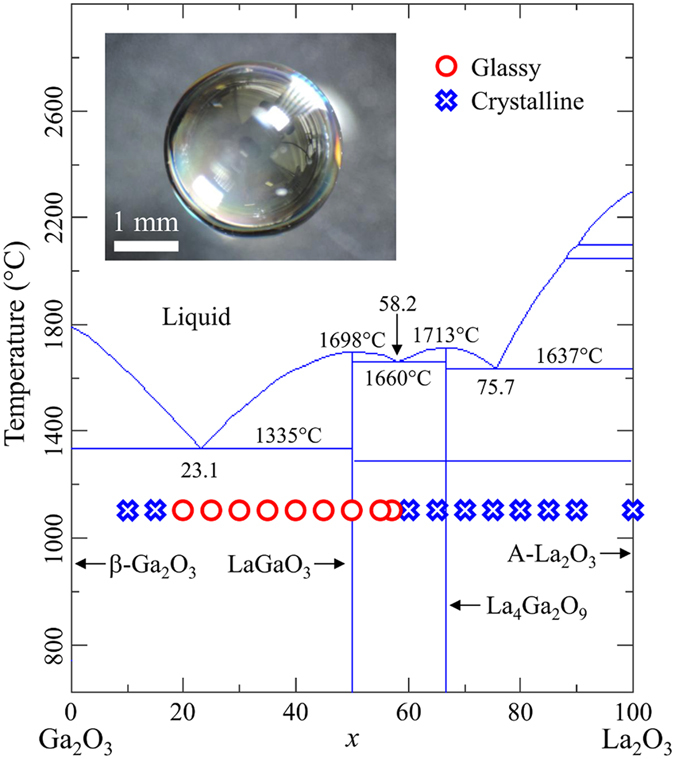
Illustration of the glass-forming region in the phase equilibrium diagram of the *x*La_2_O_3_-(100 − *x*)Ga_2_O_3_ binary system^25^ . The inset is an image of a glass sphere.

**Figure 2:**
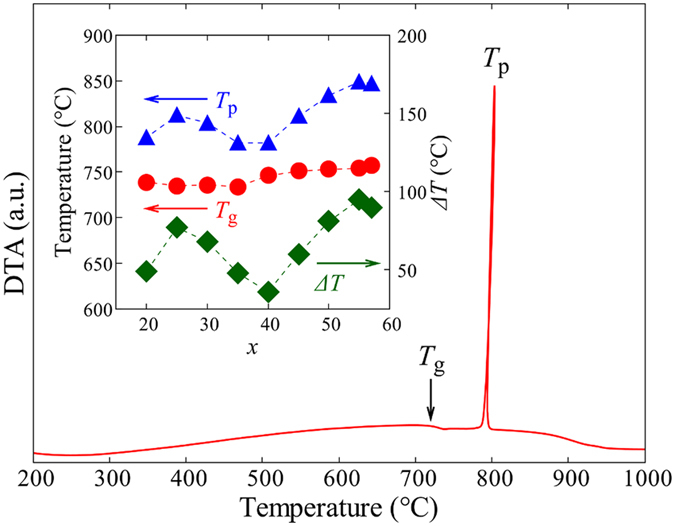
DTA curve of the 30La_2_O_3−_70Ga_2_O_3_ glass. The inset shows the compositional dependences of the glass transition temperature *T*_g_, the crystallization peak temperature *T*_p_, and the temperature gap Δ*T* (=*T*_p_ − *T*_g_). Closed circles, triangles, and squares represent *T*_g_, *T*_p_, and Δ*T*, respectively. Error bars (standard errors of 3 times measurements) are inside the symbol in each plot.

Figure 1 shows the glass-forming region of the *x*La_2_O_3_-(100 − *x*)Ga_2_O_3_ binary system on the phase equilibrium diagram^25^. The glasses were obtained at 20 ≤ *x* ≤ 57. This is a slightly wider range than that of the La_2_O_3_-Al_2_O_3_ binary system: 27–50 mol% La_2_O_3_^14^. The obtained glasses (shown in the inset of Fig. 1) were colorless and transparent spheres with diameters of 2–3 mm. At *x* = 20, 40, and 57, however, only small-sized glasses (about 1 mm in diameter) could be synthesized, and thus we carried out thermal analysis only at these compositions. Note that ICP-MS analysis confirmed that the compositional differences between experiment and theory were less than 2 mol% for all compositions, indicating that no significant deviations in the composition occurred. Figure 2 shows the DTA curve of the 30La_2_O_3_-70Ga_2_O_3_ glass. A clear glass transition and a strong exothermic peak due to crystallization were observed. The large latent heat generated during crystallization corresponds to a large energy gap between the glassy and crystalline states. The inset of Fig. 2 summarizes the compositional dependences of the glass transition temperature *T*_g_, the crystallization peak temperature *T*_p_, and the temperature gap Δ*T* (=*T*_p_ − *T*_g_). *T*_g_ showed a slight increase upon increasing *x* from 734 to 757 °C. The value of Δ*T* has been commonly used as a measure of glass stability. As can be seen in Fig. 2, all values of Δ*T* were less than 100 °C, indicating that the present glasses would be difficult to form using a conventional melt-quenching technique.

In most glasses, the glass-forming regions are close to the eutectic points^26^. The eutectic points in the La_2_O_3_-Ga_2_O_3_ system, however, located at 23.1, 58.2, and 75.7 mol% La_2_O_3_ do not correspond to the glass-forming region, as seen in Fig. 1. This situation is similar to the Y_2_O_3_-Al_2_O_3_^13^ and *Ln*_2_O_3_-Al_2_O_3_ (*Ln* = Lu, Yb, Tm, Er, Ho, Dy, Tb, Gd, and Eu) systems^14^. In these systems, some equilibrium crystalline phases are absent and metastable diagrams are dominant during the cooling of the melt. In a similar way, a possible explanation for the mismatch between the glass-forming region and the phase equilibrium diagram in the La_2_O_3_-Ga_2_O_3_ system may be the absence of the LaGaO_3_ phase in the metastable diagram, which is dominant in the actual devitrification process of the melt. In addition, it should be noted that Δ*T* took the local maximum to be at *x* = 25 or 55 and the local minimum to be at *x* = 40, as seen in Fig. 2. If only Ga_2_O_3_ and La_4_Ga_2_O_9_ were the dominant phases in crystallization, there should be only one eutectic point between these two phases and Δ*T* should take a single maximum at that point. If some crystalline phase existed in the glass-forming region, it would reduce the glass stability or prevent vitrification at the corresponding compositions^21^,^27^. Therefore, in the La_2_O_3_-Ga_2_O_3_ system, it would be expected that some crystalline phase exists at around *x* = 40, and that it forms a eutectic at around *x* = 25 and 55 with the Ga_2_O_3_ and La_4_Ga_2_O_9_ phases, respectively. Furthermore, this crystalline phase is expected to be metastable because no stable phases were observed at this composition in the La_2_O_3_-Ga_2_O_3_ phase equilibrium diagram. A detailed investigation of metastable phases in these systems will be the subject of future investigations.

**Figure 3:**
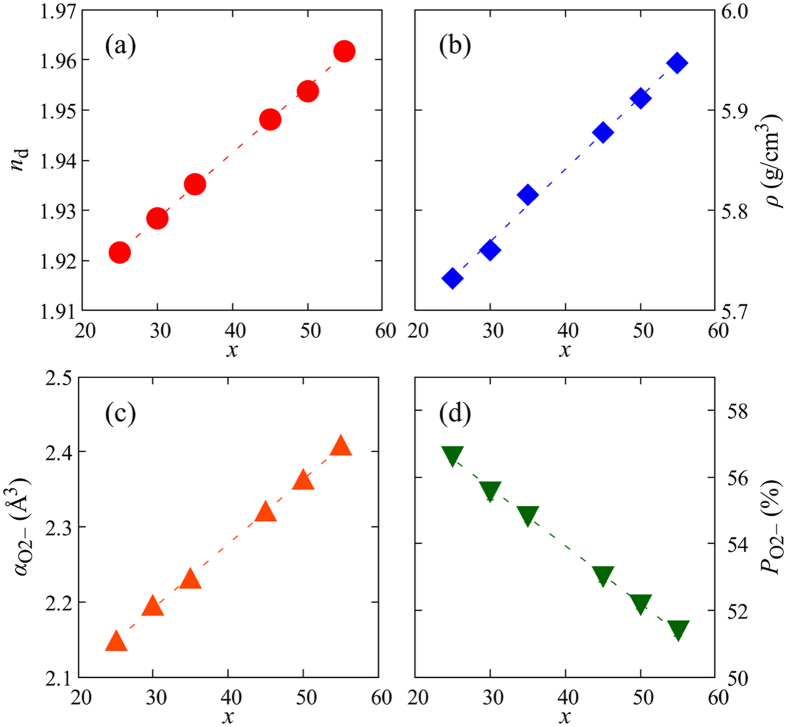
(**a**) Refractive indices *n*_d_, (**b**) densities *ρ*, (**c**) oxygen polarizabilities *α*_O2−_, and (**d**) oxygen packing densities *P*_O2−_ of *x*La_2_O_3_-(100 − *x*)Ga_2_O_3_ glasses. Error bars (standard errors of 5 times measurements in *n*_d_ and 10 times measurements in *ρ*) are inside the symbol in each plot.

Figure 3(a) and (b) show the compositional dependences of the refractive index *n*_d_ and the density *ρ* in *x*La_2_O_3_-(100 − *x*)Ga_2_O_3_ glasses, respectively. With an increase in *x, n*_d_ increased linearly from 1.921 to 1.962, and *ρ* increased linearly from 5.73 to 5.95 g/cm^3^. From the refractive index and density of the glass, the molar polarizability *α*_m_ was determined from the Lorentz–Lorenz equation.


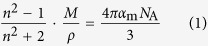


Here, *n* is the refractive index, *M* is the average molecular weight, and *N*_A_ is Avogadro’s number. For oxides, *α*_m_ can be divided into the polarizabilities of the cations and oxide ions as follows:


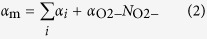


where *α*_*i*_ is the polarizability of the cation, *α*_O2−_ is the average polarizability of the oxide ions, and *N*_O2−_ is the number of oxide ions in a molecule. Using the polarizabilities of La^3+^ (1.052 Å^3^)^28^ and Ga^3+^ (0.195 Å^3^)^29^, *α*_O2−_ values were obtained and are shown in Fig. 3(c). *α*_O2−_ linearly increased from 2.16 to 2.41 Å^3^ with increasing *x*. These values are rather large and correspond to those of Bi_2_O_3_- and TeO_2_-based glasses^30^. Therefore, La_2_O_3_ should have contributed to enhancing the electron density around the oxide ions and made the glass highly ionic. The average polarizabilities of cations should also increase with *x* because the polarizability of La^3+^ is 5 times larger than that of Ga^3+^. Nevertheless, the polarization in the La_2_O_3_-Ga_2_O_3_ binary glasses was dominated by oxide ions because the polarizabilities of cations are small compared with those of oxide ions. The oxygen packing density *P*_O2−_ of the glass was calculated from the partial molar volume of oxide ions *V*_O2−_ and the ionic radius of oxygen^19^. *V*_O2−_ was obtained by subtracting the contribution of cations from the molar volume *V*_m_. Here *V*_m_ was defined as the value for the glass including 1 mol of oxide ions. The volume of 1 mol of oxide ions was calculated to be 6.92 cm^3^ using Shannon’s ionic radius (1.4 Å for O^2−^)^31^. Then, *P*_O2−_ was obtained by dividing *V*_O2−_ by 6.92 cm^3^. Figure 3(d) shows the compositional dependence of *P*_O2−_ in *x*La_2_O_3_-(100 − *x*)Ga_2_O_3_ glasses. *P*_O2−_ decreased linearly with *x* from 56.6 to 51.4%. Thus, glasses with higher Ga_2_O_3_ content have more densely packed oxide ions.

**Figure 4:**
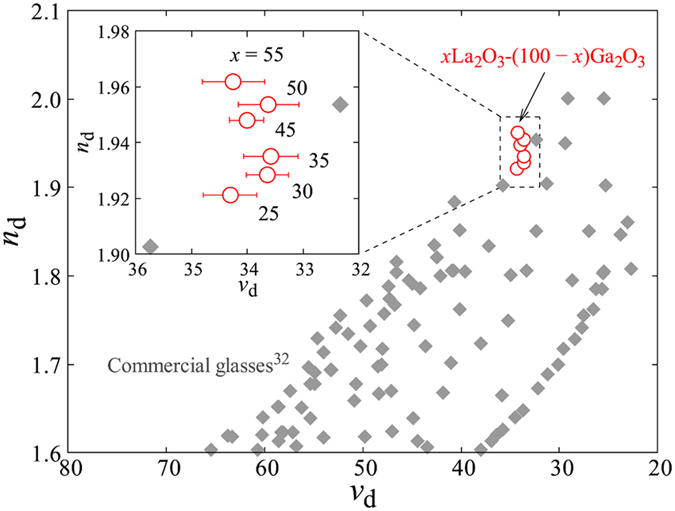
Comparison between *x*La_2_O_3_-(100 − *x*)Ga_2_O_3_ glasses and commercial optical glasses^32^ on an *n*_d_–*ν*_d_ diagram. The inset shows an enlarged view. Error bars indicate the standard errors of 5 times measurements.

Figure 4 shows a comparison between *x*La_2_O_3_-(100 − *x*)Ga_2_O_3_ glasses and commercial optical glasses^32^ on an *n*_d_–*ν*_d_ diagram. The refractive index *n*_d_ increased with *x*, whereas the Abbe number *ν*_d_ showed no significant change. Accordingly, the *x*La_2_O_3_-(100 − *x*)Ga_2_O_3_ glasses shifted to the higher *n*_d_ region and deviated gradually from the group of commercial optical glasses as *x* increased. The single oscillator model of the Drude–Voigt relation shown in following equation provides an oscillator strength, *f*, and an inherent absorption wavelength, *λ*_0_, which reflect the average features of the oscillators in the glass^33^.


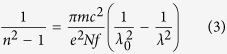


**Figure 5:**
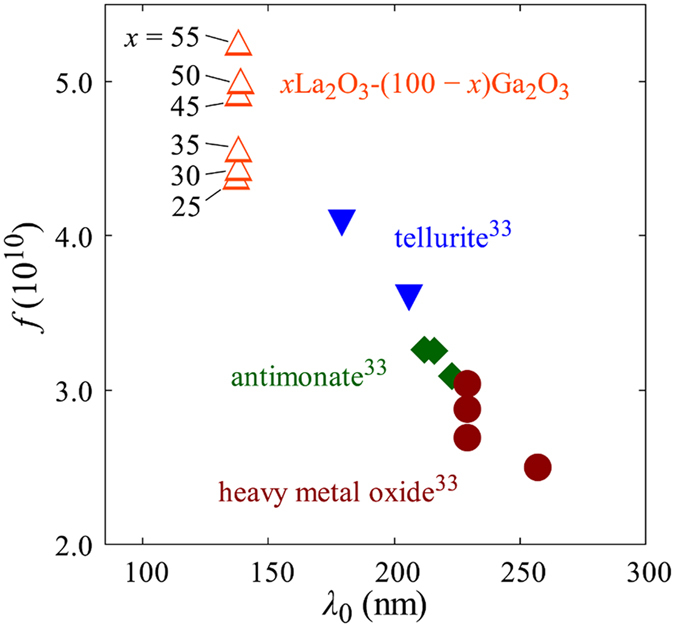
Comparison of *f* and *λ*_0_ between *x*La_2_O_3_-(100 − *x*)Ga_2_O_3_ glasses and other high refractive index glasses (tellurite, antimonite, and heavy metal oxide)^33^. Error bars (standard errors of 5 times measurements) are inside the symbol in each plot.

Here, *n* is the refractive index, *m* is the electron mass, *c* is the velocity of light in vacuum, *e* is the elementary charge, *N* is the number of molecules in a unit volume, *f* is the average oscillator strength, *λ*_0_ is the inherent absorption wavelength, and *λ* is the wavelength of light. According to this relation, a plot of (*n*^2^ − 1)^−1^ versus *λ*^−2^ is expected to be a straight line with a slope of (*πmc*^2^)/(*e*^2^*Nf*) and a *y*-axis intercept at (*πmc*^2^)/(*e*^2^*Nfλ*_0_^2^). Here, *N* can be calculated as *N* = *N*_A_*ρ/M*. Figure 5 shows the plots of the oscillator strength *f* against the inherent absorption wavelength *λ*_0_ of La_2_O_3_-Ga_2_O_3_ glasses and other high-refractive-index glasses^33^. As for the *x*La_2_O_3_-(100 − *x*)Ga_2_O_3_ glasses, *f* monotonically increased from 4.38 × 10^10^ to 5.25 × 10^10^ with increasing *x*, whereas *λ*_0_ remained at approximately 138 nm, showing no significant changes. Compared with other high-index glasses such as tellurite, antimonite, and heavy metal oxides, La_2_O_3_-Ga_2_O_3_ glasses are characterized by larger *f* and shorter *λ*_0_. This is to say that the high refractive indices of these heavy metal oxide glasses are mainly due to the longer *λ*_0_, and the increase in the refractive index of La_2_O_3_-Ga_2_O_3_ glasses with increasing La_2_O_3_ content is mainly due to the increase in *f*. This may be related to the fact that the absorption wavelength of La_2_O_3_ is much shorter than those of other heavy metal oxides such as TeO_2_, Tl_2_O, Sb_2_O_3_, PbO, and Bi_2_O_3_.

**Figure 6:**
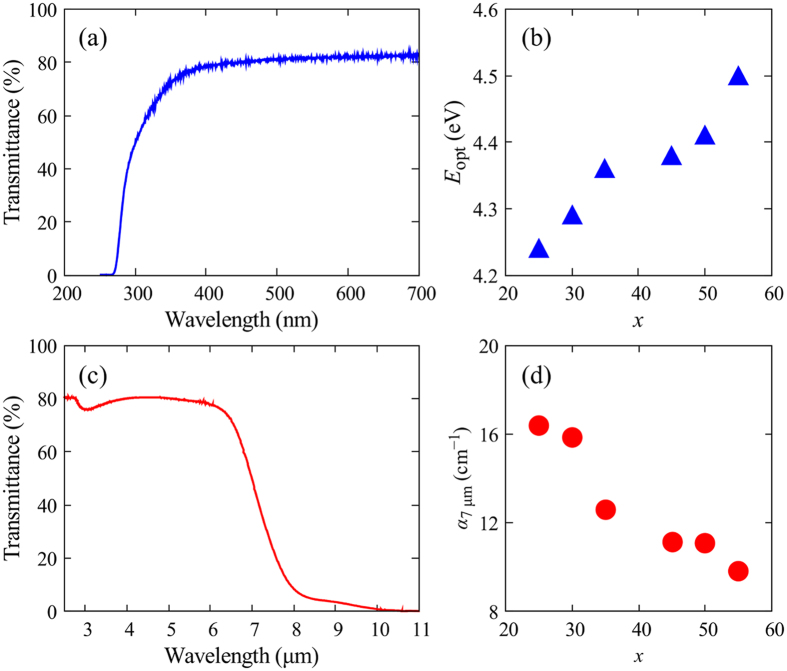
(**a**) Optical transmittance spectrum of the 55La_2_O_3_-45Ga_2_O_3_ glass in the UV–visible region, (**b**) optical energy gap, *E*_opt_, in *x*La_2_O_3_-(100 − *x*)Ga_2_O_3_, (**c**) optical transmittance spectrum of the 55La_2_O_3_-45Ga_2_O_3_ glass in the IR region, and (**d**) absorption coefficient at 7 μm, *α*_7 μm_, in *x*La_2_O_3_-(100 − *x*)Ga_2_O_3_. The sample thickness was 0.71 mm in (**a**) and (**c**).

Figure 6(a) shows the optical transmittance spectrum of the 55La_2_O_3_-45Ga_2_O_3_ glass in the UV–visible range. There was no absorption in the visible range (400–700 nm), and the absorption edge existed at 270 nm. Because of the Fresnel loss, the maximum transmittance was approximately 80%. In general, absorption edges in the UV region are caused by electronic transitions from the valence band to the conduction band, and the cut-off wavelength is determined by the energy gap between these two bands. Thus, we evaluated the optical energy gap of the glass *E*_opt_ using the Tauc equation shown as follows.


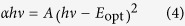


Here, *α* is the absorption coefficient, *h* is Plank’s constant, *ν* is the frequency of light, and *A* is an energy-independent constant. In addition, this equation can be rewritten as shown in following equation (5), and the optical energy gap can be determined from the discontinuity observed at a particular energy value in the d[ln(*αhν*)]/d(*hν*) versus *hν* plot.


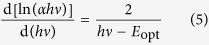


The obtained *E*_opt_ values are plotted in Fig. 6(b). *E*_opt_ increased monotonically with *x* from 4.24 to 4.50 eV. These values are much larger than those of other glasses with refractive indices of about 1.9^34^,^35^. Because both crystalline La_2_O_3_ and Ga_2_O_3_ have wide bandgaps of 5.8^36^ and 4.9 eV^37^, respectively, the large *E*_opt_ values of the La_2_O_3_-Ga_2_O_3_ glasses are convincing. In La_2_O_3_-Ga_2_O_3_ glasses, the fundamental transitions from the valence to the conduction band are expected to be O 2p → La 5d in the La–O bond and O 2p → Ga 4 s in the Ga–O bond. Considering that the energy gap in the O 2p → La 5d transition should be larger than that in the O 2p → Ga 4 s transition, the increase of *E*_opt_ in the La_2_O_3_-Ga_2_O_3_ glass is explained by the increase in the density of states of the La 5d and the O 2p orbitals with increasing *x*. The IR transmittance spectrum of the 55La_2_O_3_-45Ga_2_O_3_ glass is shown in Fig. 6(c). The transmittance was over 50% up until 7 μm, and the absorption cut-off wavelength reached over 10 μm. The slight absorption at approximately 3 μm is due to the OH groups in the glass. The cut-off wavelength of the 55La_2_O_3_-45Ga_2_O_3_ glass was much longer than those of typical oxide glasses such as silicate and borate^2^, slightly longer than those of heavy metal gallate glasses (8 μm for PbO-Bi_2_O_3_-Ga_2_O_3_^3^ and 7.5 μm for K_2_O-Ta_2_O_5_-Ga_2_O_3_ glasses^6^), and even close to those of fluoride glasses^38^. Figure 6(d) shows the plot of *α*_7 μm_, which represents the absorption coefficient of the *x*La_2_O_3_-(100 − *x*)Ga_2_O_3_ glasses at 7 μm, against *x*. The decrease in *α*_7 μm_ indicates that the IR absorption edge shifts toward longer wavelengths with increasing La_2_O_3_ content.

**Figure 7:**
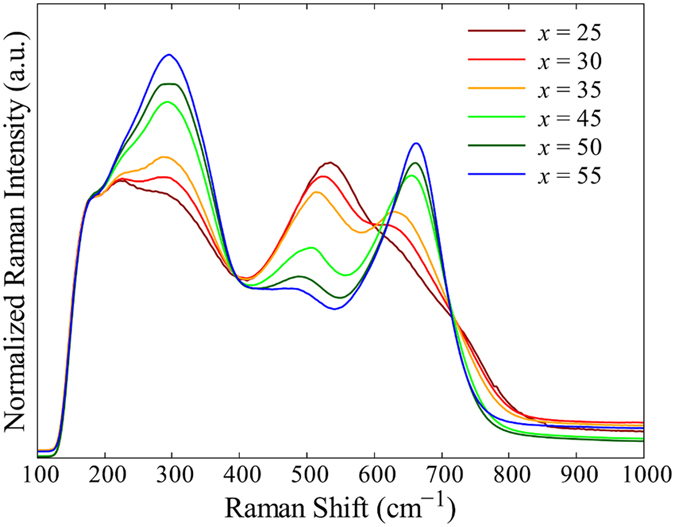
Unpolarized Raman scattering spectra for *x*La_2_O_3_-(100 − *x*)Ga_2_O_3_ glasses. The spectra were normalized at 180 cm^−1^.

Figure 7 shows the unpolarized Raman scattering spectra of *x*La_2_O_3_-(100 − *x*)Ga_2_O_3_ glasses. Three distinct bands at 300, 550, and 650 cm^−1^ were observed. The spectral shapes changed systematically with the glass compositions. With an increase in *x*, the band intensities at 300 and 650 cm^−1^ increased, whereas those at 550 cm^−1^ decreased. Detailed investigations of the Raman spectra of *R*_2_O- or *R*’O-Ga_2_O_3_ binary glasses were carried out by Fukumi and Sakka^4^. According to them, the bands at 550 and 650 cm^−1^ can be assigned to the stretching vibration of GaO_4_ tetrahedra including non-bridging oxide ions and the bending vibration of bridging oxide ions connecting two GaO_4_ tetrahedra, respectively. Furthermore, the band at 300 cm^−1^ could be assigned to the vibration of the La–O bond^39^. Therefore, the intensity ratio between the bands at 550 and 650 cm^−1^ reflects the relative quantities of bridging and non-bridging oxide ions in the glass. The Raman spectra of La_2_O_3_-Ga_2_O_3_ glasses suggested that the ratio of non-bridging oxide ions increased, whereas that of bridging oxide ions decreased when La_2_O_3_ was substituted for Ga_2_O_3_. This behavior agrees with the results obtained for the *R*_2_O- or *R*’O-Ga_2_O_3_ glasses by Fukumi and Sakka^4^. However, they also suggested that non-bridging oxide ions appeared only when the content of *R*_2_O or *R*’O was higher than 43 mol%. In La_2_O_3_-Ga_2_O_3_ glasses, the intensity of the band at 650 cm^−1^ monotonically increased with *x*, indicating that non-bridging oxide ions exist in the glasses at a much lower La_2_O_3_ content than that of *R*_2_O- or *R*’O-Ga_2_O_3_ glasses. On the other hand, Honma *et al*. evaluated the roles of La_2_O_3_ in La_2_O_3_-P_2_O_5_ binary glasses using XPS and Raman spectroscopies^28^. They showed that La_2_O_3_ acts as a network modifier, enhancing the electron density of oxide ions and increasing the ratio of non-bridging oxide ions. Similarly, in La_2_O_3_-Ga_2_O_3_ glasses, the high electron-donating ability of La_2_O_3_ would have enhanced the electron density of oxide ions and increased the ratio of non-bridging oxide ions. This is also consistent with the results of the refractive index, the average oxygen polarizability, and the oscillator strength of the La_2_O_3_-Ga_2_O_3_ glasses, as shown in Figs 3 and 5.

From the Raman spectra, it was also noticed that the maximum phonon energies of La_2_O_3_-Ga_2_O_3_ glasses were approximately 650 cm^−1^. Compared with the maximum phonon energies of other oxide glasses, such as 1100 cm^−1^ for silicate^40^, 845 cm^−1^ for germanate^41^, and 790 cm^−1^ for tellurite glasses^42^, those of La_2_O_3_-Ga_2_O_3_ glasses were significantly lower and even corresponded to the lowest of all oxide glasses. Because the absorption in the IR range is caused by the vibration of phonons, the low maximum phonon energies should be a good explanation why La_2_O_3_-Ga_2_O_3_ showed much longer IR absorption edges than other oxide glasses. From the results of this study, the extraordinarily wide transparency range from the UV to the mid-IR realized in La_2_O_3_-Ga_2_O_3_ glasses should be due to the fact that these glasses contained neither network former oxides with high phonon frequencies nor heavy metal oxides with small optical energy gaps. When rare-earth ions are incorporated as phosphors, the low maximum phonon energy of the La_2_O_3_-Ga_2_O_3_ glass is also beneficial, because the multi-phonon decay rate of rare-earth ions in a glass strongly depends on the maximum phonon energy of the host. It is, therefore, expected that the low maximum phonon energies, large solubilities of rare-earth elements, and low OH^−^ content of La_2_O_3_-Ga_2_O_3_ glasses will even enable efficient mid-IR luminescence to be achieved^38^, which cannot be obtained using typical oxide glasses with large multi-phonon relaxation rates.

## Conclusion

This paper described the successful fabrication and fundamental properties of *x*La_2_O_3_-(100 − *x*)Ga_2_O_3_ binary glasses. The glass-forming region was found to be 20 ≤ *x* ≤ 57. The refractive indices increased from 1.921 to 1.962, whereas the Abbe numbers showed no significant changes with *x*. Evaluation using the Lorentz–Lorenz relation revealed that the polarizabilities of the oxide ions were as high as 2.16–2.41 Å^3^, indicating that the glasses were highly ionic. Optical transparency measurements revealed that the absorption edges in the UV and IR regions shifted towards shorter and longer wavelengths with *x*, respectively. The optically transparent range was from 270 nm to 10 μm in the 55La_2_O_3_-45Ga_2_O_3_ glass, which corresponds to the widest range of all oxide glasses. Raman scattering spectra indicated a decrease in bridging oxide ions and an increase in non-bridging oxide ions with increasing La_2_O_3_ content. The maximum phonon energy was significantly lower (~650 cm^−1^) than that of other oxide glasses. These unique and superior characteristics suggest that La_2_O_3_-Ga_2_O_3_ glasses can be attractive host materials for various optical applications such as wideband transparent windows, achromatic lenses, or strong luminescent materials.

## Methods

High-purity La_2_O_3_ and Ga_2_O_3_ powders were mixed stoichiometrically to form *x*La_2_O_3_-(100 − *x*)Ga_2_O_3_ in a molar ratio. The mixtures were pressed into pellets under a pressure of 20 MPa and sintered at 1200 °C for 12 h in an air atmosphere. Pieces of approximately 50 mg taken from the broken pellets were vitrified in an aerodynamic levitation furnace. The specimens were levitated by an O_2_ gas flow and heated by CO_2_ lasers. A high-resolution, charge-coupled device camera was used to observe the levitated samples. Glass formation was confirmed by Cu *K*α X-ray diffraction measurements. The compositions of the glasses were investigated by inductively coupled plasma–mass spectrometry (ICP-MS) analysis (Agilent Technologies Agilent 7700x).

*T*_g_ and *T*_p_ were determined by differential thermal analysis (DTA) in an air atmosphere at a heating rate of 10 °C/min (Rigaku Thermo plus EVO2 TG8121). All glasses were annealed at a temperature near *T*_g_ for 10 min in order to remove thermal strain prior to the physical property measurements. The densities of the glasses were measured using a gas pycnometer (Micromeritics AccuPycII 1340). For the optical measurements, the glasses were sliced and polished into disks approximately 1 mm thick. The refractive indices were measured using a prism coupler (Metricon Model 2010/M) at wavelengths of 473, 594.1, and 656 nm. The measured indices were fitted by the Drude–Voigt model shown in equation (1) using the least squares method, and the refractive indices and the abbe numbers were calculated from the fitted curves. The optical transmittance spectra were taken in the wavelength range of 250–700 nm using a UV-vis-NIR spectrophotometer (Hitachi High-Technologies UH4150). The IR transmittance spectra were taken in the wavenumber range of 400–4000 cm^−1^ using a Fourier transform infrared spectrophotometer (Thermo Fisher Scientific Nicolet 6700). Unpolarized Raman scattering spectra were taken using a micro-laser-Raman spectrometer (JASCO NRS-7100) with excitation provided using an Ar^+^ laser at 488 nm.

## Additional Information

**How to cite this article:** Yoshimoto, K. *et al*. Low phonon energies and wideband optical windows of La_2_O_3_-Ga_2_O_3_ glasses prepared using an aerodynamic levitation technique. *Sci. Rep.* **7**, 45600; doi: 10.1038/srep45600 (2017).

**Publisher's note:** Springer Nature remains neutral with regard to jurisdictional claims in published maps and institutional affiliations.
